# Selectivity and anti‐tumor immune elevation by vascular‐targeted photodynamic therapy of mouse orthotopic bladder cancer model

**DOI:** 10.1111/php.14048

**Published:** 2025-03-06

**Authors:** Jie Chen, Natalia Kudinova, Rebecca Dubrovsky, Jasmine Thomas, Karan Nagar, Lucas Nogueira, Avigdor Scherz, Kwanghee Kim, Jonathan Coleman

**Affiliations:** ^1^ Department of Surgery Memorial Sloan Kettering Cancer Research Center New York New York USA; ^2^ Department of Plants Science Weizmann Institute of Science Rehovot Israel; ^3^ Urology Service, Department of Surgery Memorial Sloan Kettering Cancer Research Center New York New York USA

**Keywords:** anti‐tumor immunity, bladder cancer, orthotopic mouse model, vascular‐targeted photodynamic therapy

## Abstract

Vascular‐targeted photodynamic therapy (VTP) with WST11 is a non‐surgical tumor ablation approach that is currently being tested in a phase 3 clinical trial for the treatment of upper tract urothelial cancer. WST11‐VTP utilizes illumination, leading to hypoxia, and production of free radicals followed by coagulative necrosis. Here, we tested the hypothesis that WST11‐VTP can safely ablate muscle‐invasive MB‐49‐ luc bladder tumors in an orthotopic mouse model while sparing the surrounding normal tissue. For the safety study, normal mouse bladders were WST11‐VTP treated. Fourteen days post‐VTP granulomas in local areas around the ablation zone were noticed, which recovered after 44 days. MB49‐luc orthotopic tumors at the muscle‐invasive stage appeared to be effectively ablated by VTP 4–10 days post‐treatment. The anti‐tumor response was reflected in the increased invasion of CD4^+^, CD8^+^ T cells, myeloid CD11b^+^ cells, and NK cells in tumor tissue at 7 days post‐therapy. Moreover, VTP therapy prolonged the survival of mice bearing orthotopic tumors compared with the untreated control. These results suggest that VTP can selectively ablate malignant tumors in the bladder and promote a robust anti‐tumor response in a mouse model that can further augment the therapeutic outcome.

## Graphical abstract

Vascular‐targeted photodynamic therapy (VTP) using the photosensitizer WST11 is a non‐surgical tumor ablation approach. Light activation of WST11 leads to excessive generation of reactive oxygen species, which causes tumor vascular arrest, followed by destruction of tumor blood vessels and tumor necrosis. WST11‐VTP can safely ablate muscle‐invasive MB‐49‐ luc bladder tumors in an orthotopic mouse model and promote an anti‐tumor immunity boost while preserving normal bladder tissue that can further augment the therapeutic outcome.

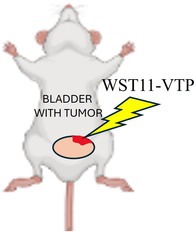

**Abbreviations**
BC: bladder cancer
BCG: Bacillius Calmette‐Guerin
Inj: injected
IVIS: in vitro imaging system
NMIBC: non‐muscle invasive bladder cancer
NO: nitric oxide
NS: 0.9% sodium chloride
TME: tumor microenviroment
VTP: vascular targeted therapy

## INTRODUCTION

Bladder cancer (BC) is the fourth most common cancer in men and less common in women. By 2024, approximately 63,070 men and 20,120 women are expected to be diagnosed with BC in the United States, resulting in 16,840 deaths. Approximately 9 of 10 people with this type of cancer are over the age of 55 years, with a mean age of 73 years.^1^ Most BCs (75%) are diagnosed at an early stage (non‐muscle‐invasive [NMIBC]) and generally have a good prognosis with a 20%–30% rate of progression. The standard procedure for NMIBC is transurethral resection of bladder tumor, with or without additional intravesical (into the bladder lumen) administration of Bacillus Calmette‐Guerin (BCG) or mitomycin C.^2^, ^3^, ^4^ Unfortunately, NMIBC has a high recurrence rate. Vascular‐targeted photodynamic therapy (VTP) is a noninvasive option for preventing or delaying surgery in BCG‐resistant patients. VTP targets the tumor vasculature through photoactivation by the water‐soluble photosensitizing agent WST11 (Padeliporfin, TOOKAD). Following intravenous administration, WST11 binds non‐covalently to albumin and is sequestered within the circulation, with a half‐life of <60 min in humans, thus avoiding long‐term skin toxicity. Excessive generation of reactive oxygen species upon light activation of WST11 confirms immediate hypoxia, which activates nitric oxide (NO) radical generation. The co‐generation of nitric oxide and oxygen radicals results in the generation of peroxynitrite, which causes tumor vascular arrest, followed by tumor necrosis, and eradication within 48 h.^5^ Moreover, VTP induces a long‐lasting immune response in cellular and humoral components.^6^ WST11 (Padeleporfin) VTP has been approved in Europe, Israel, and Mexico as a first‐line treatment for men with low‐risk prostate cancer (Studies NCT01310894 and NCT01875393).^7^ Currently, it is under evaluation in phase III clinical trials for the treatment of upper tract urothelial carcinoma (Study NCT04620239).^8^ In the present study, we aimed to preclinically test the feasibility of WST11‐VTP for treating tumors of the lower urinary tract, such as muscle‐invasive BC. To this end, we constructed an orthotopic mouse model in which the immune profile evolved within the tumor microenvironment (TME), which is expected to mimic human tumors.

## MATERIALS AND METHODS

### Animals

C57BL/6 male mice (12–16 weeks) were purchased from Jackson Laboratory (USA). The weight of the mice was 30 ± 3 g. All animal experiments were approved by the MSKCC Institutional Animal Care and Use Committee (IACUC).

### VTP on normal mouse bladder

Mice were anesthetized with 2% isoflurane inhalation. The painkillers meloxicam (0.1 mg/kg) and buprenorphine (0.015 mg/kg) were subcutaneously administered before treatment. Anesthetized mice were placed on a warm plate in the supine position for all the surgical procedures. A midline abdominal incision (1 cm) was made followed by bladder exposure. In this safety study, 22 mice were used, of which 19 mice were injected with 100 μL of 0.9% sodium chloride (NS) through the bladder wall into the bladder lumen 1 week before treatment and then randomly divided into four groups: NS group received only NS injection (NS inj), NS + WST11 group received NS injection and retro‐orbital injection of 9 mg/kg WST11, NS + laser group received NS injection accompanied with bladder illumination with laser, and NS + VTP group received NS injection accompanied with VTP treatment. The non‐treatment group (sham) consisted of three mice, and only an abdominal incision was made. The VTP protocol included retro‐orbital injection of 9 mg/kg of WST11 (Steba Biotech, Luxemburg) with a lag time of 2 min (drug/light interval) before illumination of the entire bladder. Illumination of the bladder was performed with frontal fibers (Medlight, Switzerland) at a light power of 85 mW/cm at 753 nm provided by a medical laser (Modulight, Finland) for five intermittent illumination sessions, each lasting 30 s. At the end of the follow‐up experiments, mice were euthanized with CO_2_, and bladders were collected and processed for histology (hematoxylin& eosin [H&E] trichrome staining), immunohistochemistry with CD11b and CD68 antibodies, and TUNEL staining.

### Histology and immunohistochemistry

After excision, the bladder was fixed in 10% neutral buffered formalin (Fisher Scientific, USA). Specimens were embedded in paraffin and sections were stained with hematoxylin and eosin (H&E; Sigma‐Aldrich, USA) using standard procedures. Antibodies against CD31 (ab182981), CD4 (ab183685), and CD11b (ab13335) were purchased from Abcam (Cambridge, USA). CD8 (#98941) and CD56 (#99746) cells were obtained from Cell Signaling Technology (USA). CD68 (PA1518) was purchased from Boster Biological Technology, USA. TUNEL (terminal transferase, #03333566001, Biotin‐16‐dUTP, #11093070910) was purchased from Roche (Basel, USA). Trichrome stain (HT‐10516) was obtained from Sigma‐Aldrich (USA).

### Orthotopic bladder model development

The MB‐49 cells (Applied Biological Materials Inc., USA) were cultured in Dulbecco's modified eagle medium (DMEM) supplemented with 50 μg/mL  streptomycin (Invitrogen, USA), 50 units/mL penicillin (Invitrogen, USA), and 10% fetal bovine serum (FBS) (Life Technologies, Grand Island, NY, USA). Murine stem cell virus puromycin–luciferase–GFP was transfected into GP2‐293 pantropic retroviral packaging cells (BD Biosciences, San Jose, CA, USA) using lipofectamin 2000 (Invitrogen, Grand Island, NY, USA), and the collected retrovirus was used to infect cells from the MB‐49 mouse BC cell line. Infected cells were selected with 0.5 μg/mL puromycin (Invitrogen) and the surviving pool of cells was designated as MB‐49‐luc. Infection was carried out in the presence of 6 μg/mL polybrene (Sigma, St. Louis, MO, USA). Cells were maintained in a humidified incubator (NuAire Inc., USA) with 5% CO_2_ at 37°C. Cultured murine BC cells were collected from culture plates using Tryple Express (Invitrogen, USA). The cells were then suspended at 1 × 10^6^ cells/ml in complete DMEM on ice prior to orthotopic transplantation. An orthotopic animal model was established using a previously described method.^9^ Briefly, the mice were anesthetized with 2% isoflurane inhalation. Painkillers (0.1 mg/kg of meloxicam and buprenorphine, 0.015 mg/kg) were subcutaneously injected. Anesthetized mice were placed in a supine position on a warm plate for all surgical procedures. A midline abdominal incision (1 cm) was made followed by bladder exposure. The cells (1 × 10^5^/100 μL) were injected through the dome bladder wall into the bladder lumen. The abdominal wall was closed by suture (5–0 Vicryl absorbable, Ethicon, USA) followed by 9 mm autoclips (Braintree Scientific, USA) to close the skin. Mice were kept under anesthesia for 1 h and then returned to their cages for recovery. Mice were restricted to drinking water for 1 h during the recovery period.

To evaluate tumor growth, the bladders were harvested 3, 4, and 6 days after implantation and subjected to H&E staining and immunostaining with antibodies against CD31. In some experiments, tumor growth was monitored using in vitro imaging system (IVIS) (Spectrum SAI Core Optical Imaging, USA). Briefly, mice were anesthetized by inhalation of 2% isoflurane and retro‐orbitally injected with 100 mg/kg D‐luciferin solution (Invitrogen, USA). Three minutes after the injection, the animals were placed in the IVIS chamber in a standardized manner. Images based on luciferin activity were acquired for 10 s with the mice maintained under general anesthesia via a nasal cone.

### VTP on orthotopic bladder model

VTP treatment was performed 6 days after implantation when the presence of bladder tumors was confirmed. Mice were anesthetized using 2% isoflurane inhalation. Painkillers (0.1 mg/kg of meloxicam, 0.015 mg/kg buprenorphine) were administered subcutaneously. Anesthetized mice were placed on a warm plate in the supine position for all the surgical procedures. A midline abdominal incision (1 cm) was made followed by bladder exposure. In this study, mice were divided into treated and untreated control groups. In the treatment group, 9 mg/kg WST11 was retro‐orbitally administered. Two minutes after injection, entire bladders were illuminated with a frontal fiber (Medlight, Switzerland) at a light power of 85 mW/cm (Modulight, Finland) in five consecutive cycles with 30‐s interval cycles for a total of 2.5 min (5 × 30 s) intermittently. The untreated control group received an injection of 100 μL 0.9% sodium chloride (NS) through the bladder wall into the bladder lumen on the same day that the treated group underwent tumor implantation.

To evaluate the efficacy of VTP, mouse bladders from all groups were harvested 4–10 days after VTP treatment. The bladders were weighed and processed for histology and immunohistochemistry, using antibodies against CD8, CD4, CD11b, and CD56. For survival experiments, the untreated and VTP groups were checked for healthy signs until 36 days after VTP treatment. The bladders were harvested, fixed in formalin, and processed for H&E and Trichrome staining.

### Statistical analysis

Data are presented as the mean ± SEM, and differences between groups were calculated using the unpaired two‐tailed *t*‐test. Log‐rank and Gehan‐Breslow‐Wilcoxon tests were used to compare differences in overall survival. Statistical analysis was performed using the Prism 8.2 software (GraphPad Software, Inc. USA).

## RESULTS

### VTP on normal mice bladders: safety study

**FIGURE 1:**
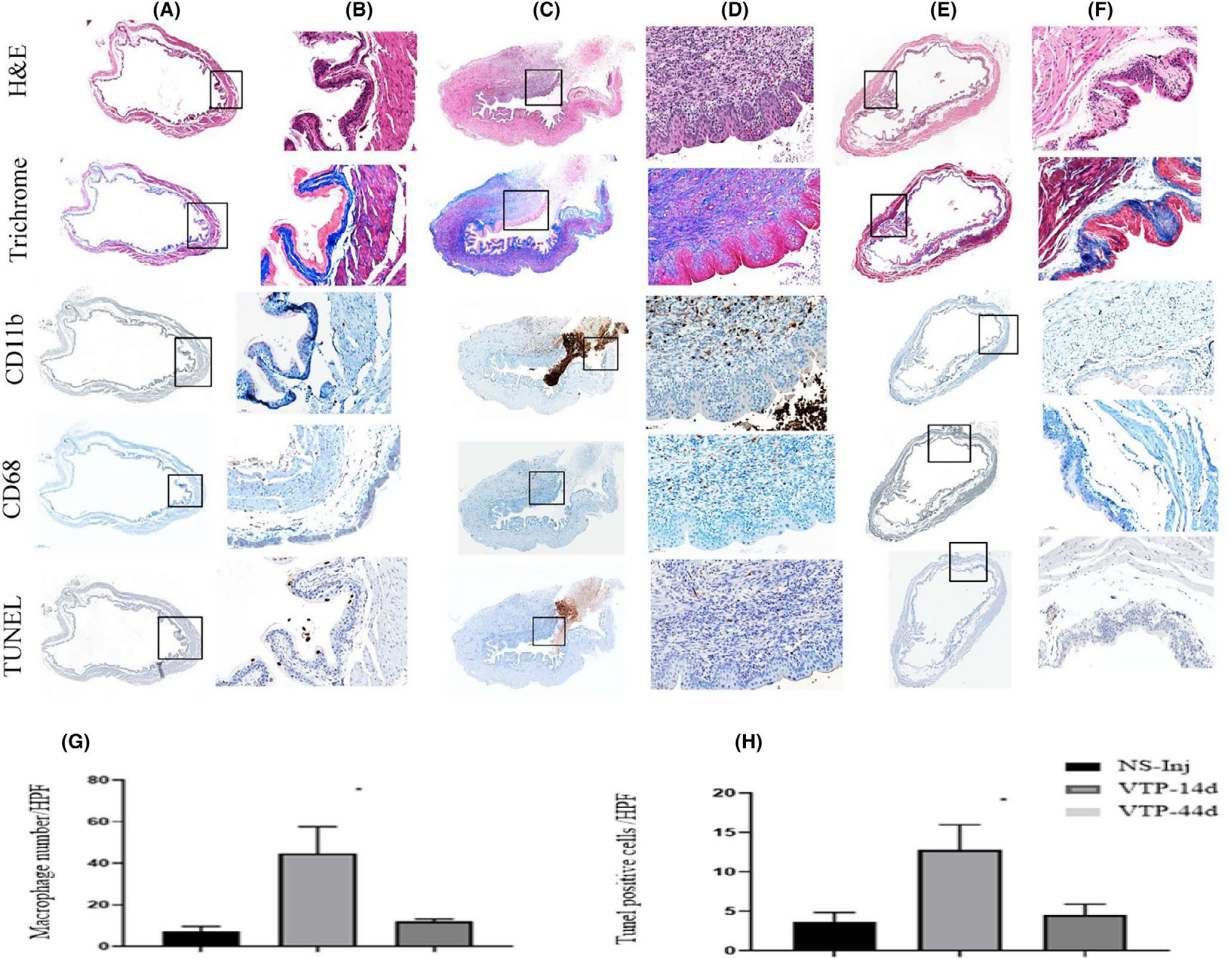
Histopathology and immunohistochemistry of normal bladder after VTP. (A) NS‐injected controls (4× magnification); (B) NS‐injected controls (20×); (C) VTP treated at 14 days post‐treatment (4×); (D) VTP treated at 14 days post‐treatment in (20×); (E, F) VTP treated at 44 days post‐treatment in 4× and 20×, respectively. Scale bars: Blue (100 μm) and green (500 μm). Quantification (high power fields, HPF) of TUNEL positive cells (G) and macrophages (H) NS inj group and VTP groups 14 and 44 days (*p < 0.02).

To investigate the effect of VTP on normal bladders, the mice were randomly divided into five groups: four controls and one treatment group. The control groups included: (1) abdominal surgery only (sham), (2) surgery and injection of 0.9% saline into the bladder lumen (NS injected), (3) surgery and injection of 0.9% saline into the bladder lumen followed by retro‐orbital injection of 9 mg/kg WST11 (NS + WST11, dark control), and (4) surgery and injection of 0.9% saline into the bladder lumen followed by laser illumination (NS+ laser, light control). The treatment group underwent surgery and retro‐orbital injection of 9 mg/kg WST11, followed by laser illumination of the bladder (VTP group). Fourteen days after treatment, the bladders of the VTP‐treated and control groups were excised and subjected to histological analysis. Twelve animals from the VTP group were kept for 44 days post‐treatment to evaluate the long‐term effects. None of the control groups showed visible changes in histological evaluation. Representative histological sections of the NS‐injected group are shown in Figure 1A,B. Fourteen days post‐treatment, 50% of the animals in the VTP group demonstrated hemorrhage, edema, and granulomas (Figure 1C,D; H&E) with fibrosis, which was confirmed by trichrome staining (Figure 1C,D [Trichrome]). The remaining 50% of the animals showed no bladder damage. The histological samples from the VTP group (14 days) and NS‐injected group were stained with TUNEL, CD11b, and CD68 antibodies to investigate the inflammatory effect of VTP on normal bladder tissue. In the VTP group, compared to the NS‐injected control invasion of myeloid cells and macrophages was observed in all bladder samples (Figure 1C,D; CD68 and CD11b, respectively). Apoptotic processes using TUNEL assay were observed in the ablation zone in all bladder samples in the VTP group (Figure 1C,D). On Day 44 post‐VTP, 50% of VTP bladder samples showed no bladder damage, as confirmed by hematoxylin and eosin and trichrome staining (Figure 1E,F; H&E, Trichrome). A focal area of fibrosis was observed in 20% of animals, with no bladder damage. In 20%–30% of bladder samples, granulomas with fibrin deposition and myeloid cell infiltration were still present. The infiltration of macrophages and the number of apoptotic cells at this time point were reduced to the control levels (Figure 1G,H, respectively). The bladder function was intact, and all animals in the VTP group had normal urination. These findings indicate that normal bladder tissue after VTP could recover in the long term after VTP.

### Establishment of orthotopic bladder model

To develop the orthotopic bladder mouse model, 10^5^ MB49‐luc cells were injected into the bladder lumen of the mice in the modeling group. The mice in the untreated control group underwent sham surgery under the same conditions.

**FIGURE 2:**
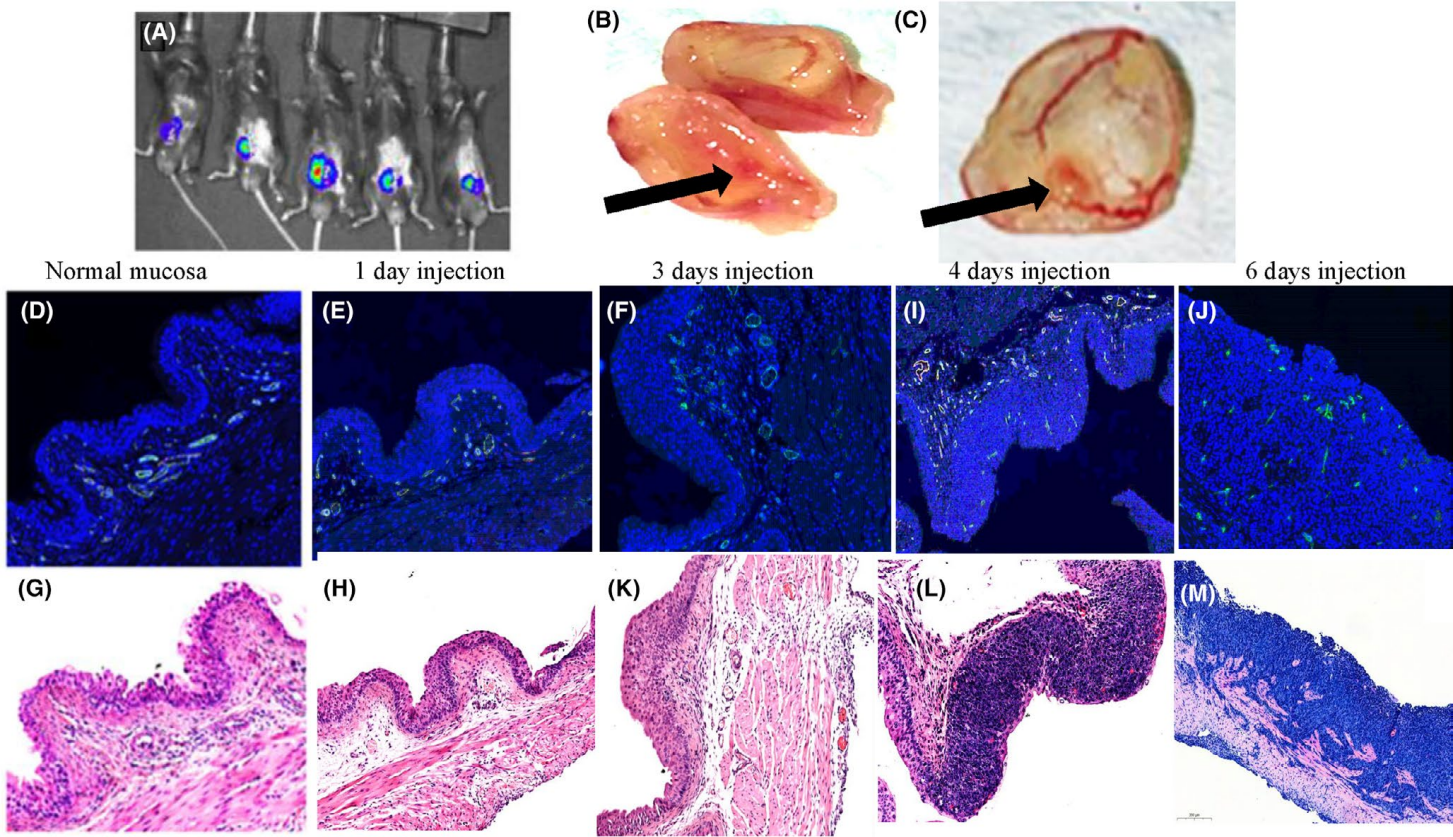
Evaluation of MB49‐ luc orthotopic bladder model. The presence of MB49‐Luc tumor in the bladder wall was confirmed by IVIS (A) or gross pathology (B and C) at 3 and 4 days post‐implantation, respectively. Expression of CD31^+^ endothelial cells marker in bladder mucosa indicates tumor growth: (D) control bladder (E, F, I, J) 1, 3, 4, and 6 days post‐implantation, respectively. H&E staining: (G) control bladder, (H, K, L, M) 1, 3, 4, and 6 days after implantation. Scale bars: Blue(100 μm), green (500 μm).

Mice from the modeling group displayed the first luciferase signal 3 days after tumor cell administration (Figure 2A). The tumors were visualized on the bladder wall (Figure 2B), corresponding to the histological findings of a few foci of tumor cells in the bladder mucosa (Figure 2K). Visualization of the tumor was more pronounced 4 days post‐inoculation (Figure 2C), and tumor cells were observed in the mucosal and submucosal layers (Figure 2L). On Day 6 post‐inoculation, the tumor cells had spread to the muscle layer (Figure 2M). No tumor cells were observed in the modeling group 1‐day post‐tumor instillation (Figure 2H). The normal bladders in the control (sham) group are shown in Figure 2G.

The main target of VTP is the tumor vasculature. Tumor growth was monitored by measuring CD31 expression. CD31 is known as platelet endothelial cell adhesion molecule 1 (PECAM‐1) and is thought to be a specific marker of vascular differentiation.^9^, ^10^ Neither control (sham) bladders nor bladders from the modeling group at 1 and 3 days post‐inoculation showed CD31 expression (Figure 2D–F, respectively). The expression of CD31 in the bladder mucosa was noticed at 4 days and continued until 6 days after tumor cell administration (Figure 2I,J, respectively). This data indicate that tumors with newly formed blood vessels were established in the bladder mucosa as early as 4 days post‐inoculation. VTP treatment of tumor‐bearing bladders was performed 6 days post‐inoculation when the tumors invaded the muscle layer and formed new blood vessels.

### VTP on orthotopic bladder model: efficacy and survival study

**FIGURE 3:**
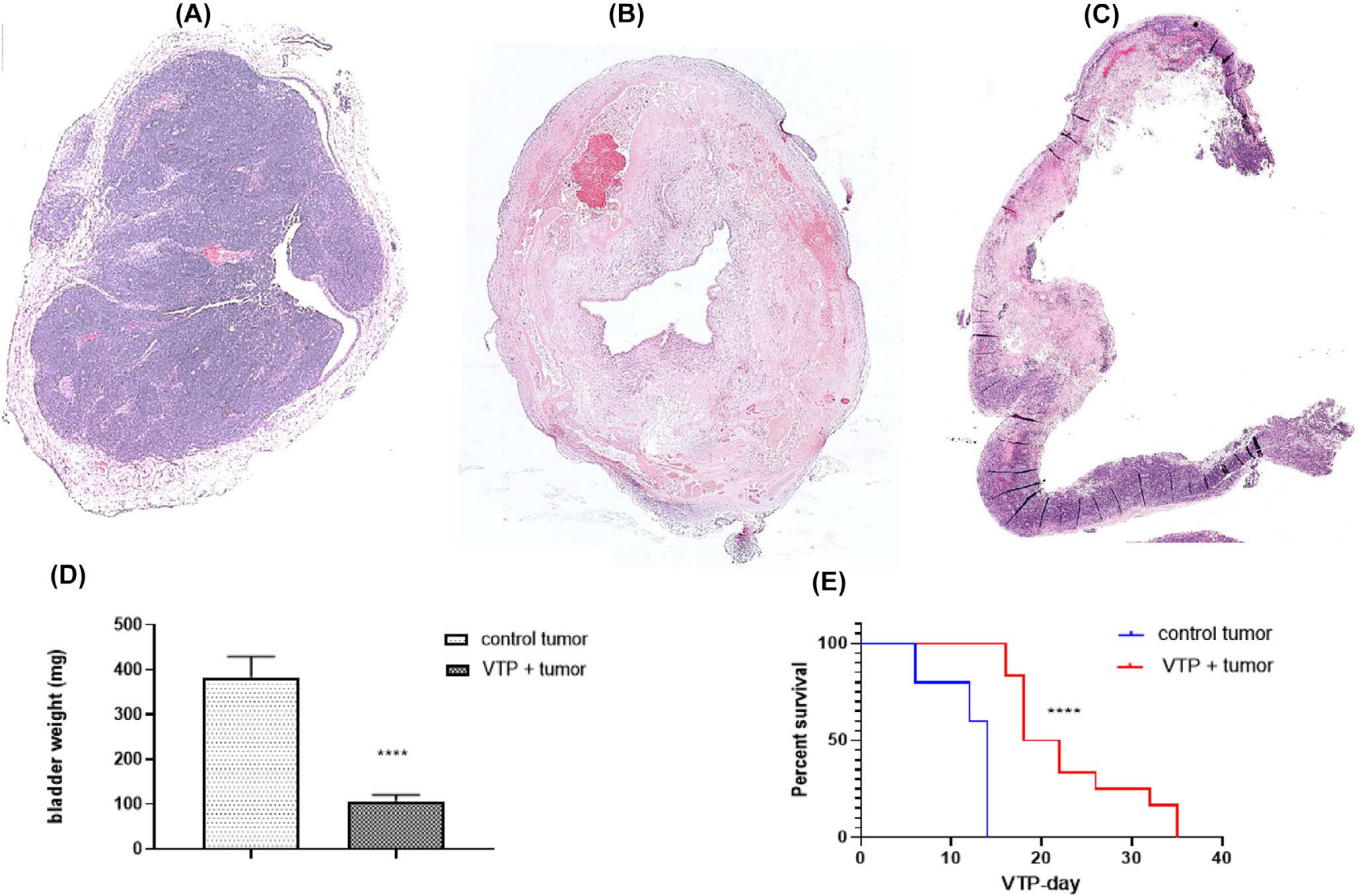
VTP effect on MB49‐luc orthotopic bladder carcinoma. Histological evaluation of VTP therapy (A) Untreated control bladder 10 days after MB49‐ luc implantation, (B, C) VTP ablation of the entire bladder bearing Mb49‐luc tumor at 10 and 36 days post‐treatment, respectively (D) bladder weight of untreated control and VTP groups at 10 days post‐treatment (****p < 0.0001), (E) overall survival of MB49‐luc tumor‐bearing mice from treatment and untreated groups. Survival significance was determined using the log‐rank test (****p < 0.0001). Scale bar: 100 μm.

VTP was performed on tumor‐bearing bladders on Day 6 post‐grafting. To study the efficacy of VTP, bladders bearing tumors from both the untreated control and treated groups were harvested at 4, 6, 7, 8, 9, and 10 days post‐treatment. Representative histological H&E‐stained images are shown in Figure 3. Figure 3B shows the bladder 10 days post‐VTP with necrosis, hemorrhage, and neutrophilic inflammation in the bladder wall. The corresponding untreated control for viable tumors is shown in Figure 3A. In periods of 4–7 days and 8–10 days post‐VTP, 50% and 54% of the mice, respectively, showed >95% tumor/bladder ablation. Moreover, the weight of the tumor‐bearing bladders in the treatment group on Day 10 post‐VTP was significantly lower (*p* < 0.0001) than that of the corresponding untreated control bladders (Figure 3D). Follow‐up of the untreated control and VTP groups continued until Day 36 post‐VTP, when the last animal in the treatment group had to be sacrificed because of life‐threatening conditions (Figure 3E). VTP significantly prolonged the survival of tumor‐bearing animals (*p* < 0.0001). Histologically, tumor cells were observed in all animals in the VTP group, indicating incomplete response or recurrence after VTP (Figure 3C).

### VTP on the orthotopic bladder model enhances the immune response

**FIGURE 4:**
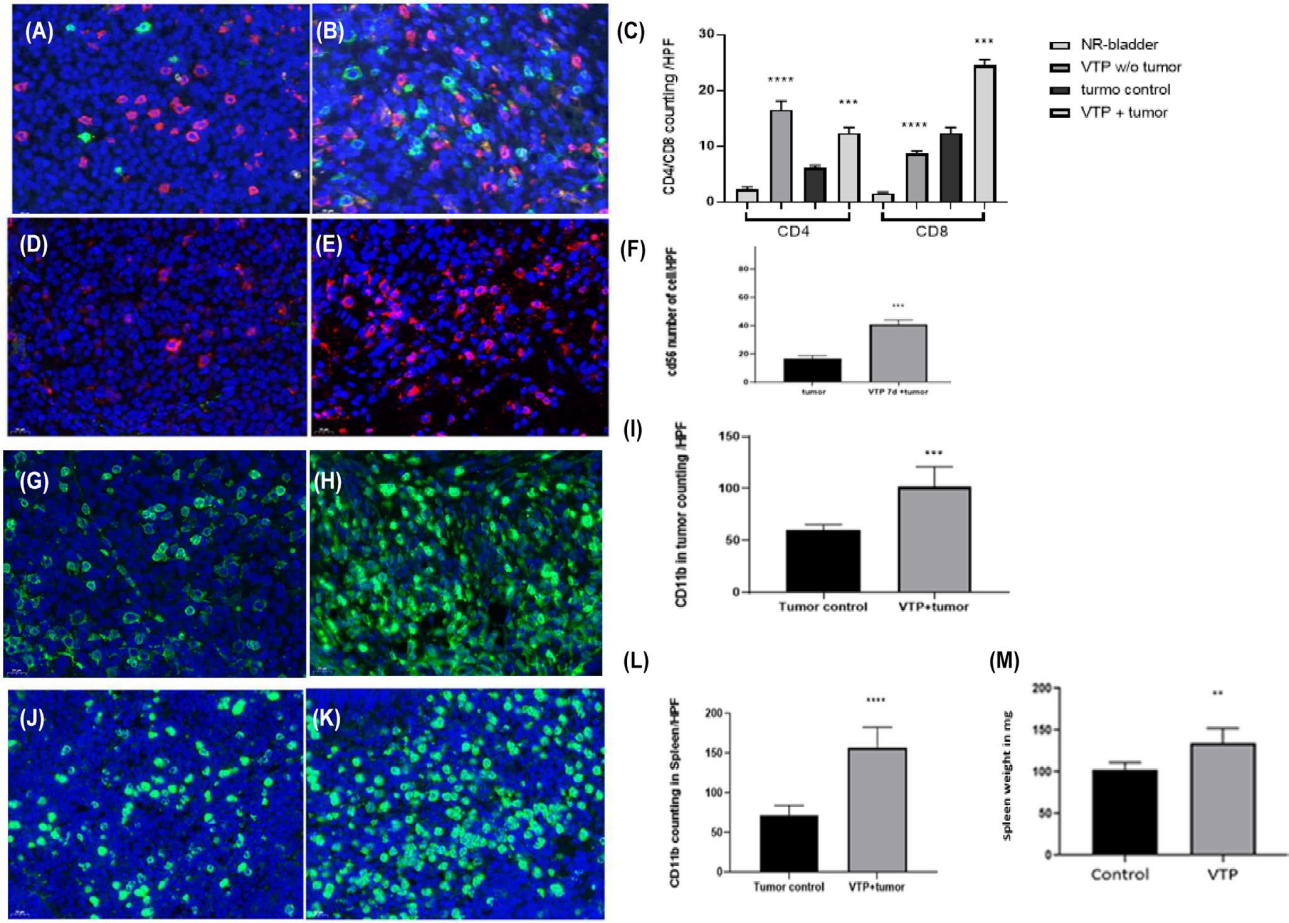
Effect of VTP on infiltration of immune cells into MB49‐luc tumor and spleen. Immunostaining fluorescent images (CD4^+^ [green] and CD8^+^ [red]). (A, B) Infiltration of CD8^+^ and CD4^+^ T cells in untreated control and post‐VTP tumors, respectively; (C) Quantification of CD4^+^ and CD8^+^ T cells in treatment groups; (D, E) CD56^+^ NK cell invasion in untreated control and post‐VTP tumors, respectively; (F) Quantification of NK cells in treatment groups; (G, H) Infiltration of CD11b^+^ myeloid cells in untreated control and post‐VTP tumors, respectively; (I) Quantification of CD11b^+^ myeloid cells in treatment groups; (J, K) Infiltration of CD11b^+^ myeloid cells in untreated control and post‐ VTP spleen, respectively; (L) CD11b^+^ counting in spleen of treatment groups; (M) Spleen weight (mg) in untreated control (*n* = 5) and VTP groups (*n* = 12) (statistical significance: **p < 0.0024, ***p < 0.0004, ****p < 0.0001).

An integral part of the WST11‐ VTP‐mediated tumor eradication mechanism is the induction of an immune response.^6^, ^11^ In the present study, we compared T cell and NK cell recruitment between the untreated control and treatment groups on Day 7 post‐VTP. The untreated control tumors contained a few CD4, CD8, and NK cells (Figure 4A,D, respectively). Immunohistochemistry on Day 7 after treatment confirmed the presence of high numbers of CD4 and CD8 T lymphocytes (Figure 4B,C), as well as increased invasion of NK cells (Figure 4E,F) in the VTP‐treated tumors. The spleen weight was significantly higher in the VTP‐treated group (Figure 4M). CD11b antibodies were used to detect myeloid cells. Figure 4H,I show a significant increase in the infiltration of myeloid cells into the tumor microenvironment after VTP (Figure 4H,I), corroborating their elevation in the spleen (Figure 4K,L) on Day 7 post‐VTP compared with the untreated control (Figure 4G,J, respectively). These observations confirmed that CD4 T cells, CD8 T cells, NK cells, and myeloid cells were recruited to MB49 orthotopic bladder tumors in response to VTP. Moreover, VTP therapy led to an influx of myeloid cells into the spleen.

## DISCUSSION

BC was the first cancer for which PDT was approved in 1993. However, PDT with hematoporphyrin results in side effects such as severe skin photosensitization due to prolonged clearance, bladder wall fibrosis, and bladder contraction.^12^, ^13^ WST11‐VTP differs from classical PDT in its mechanism of action and offers many advantages, including low or practically no skin phototoxicity due to rapid clearance. Important features of WST11 VTP are (i) differential response of normal vs. tumor tissue to the treatment protocol and (ii) induction of intensive innate and adaptive anti‐tumor immunity that complements tumor tissue eradication. To date, WST11‐VTP has been predominantly applied in preclinical and clinical treatments using continuous illumination at 753 nm for 10–20 min immediately after WST11 administration.^14^ This regimen has been established in numerous animal models, including MB‐49, in the subcutaneous setting, and has been applied in the clinical arena. As the tumor microenvironment plays a significant role in the response to treatment, several indications have been studied in orthotopic animal settings. However, in a preliminary study, we found that the current treatment regimen was too toxic in an orthotopic mouse model of BC. However, intermittent light application was tolerated by the mice, allowing us to answer two fundamental questions: (i) Is there a different response between normal and tumor tissues in the bladder environment? (ii) Is VTP followed by an anti‐tumor immune response when applied to the target organ? Previously, it was demonstrated that VTP induced tissue ablation in the porcine liver and kidney while preserving critical organ structures.^15^ Murrel et al. showed that WST11‐VTP can be successfully and safely applied to the porcine ureter and renal pelvis to produce superficial or deep ablation.^16^ In the present study, we used WST11‐VTP in normal mouse bladders to investigate the effects of treatment on normal tissues. On Day 14 post‐treatment, an acute response to WST11‐ VTP was still visible in half of the normal bladders. This response includes hemorrhage, edema, granulomas, and inflammation, which are reflected in myeloid and macrophage cell invasion. One month later (44 days post‐VTP), granulomas with fibrin deposition and myeloid cell invasion were still present in 20%–30% of the animals. The remaining 80%–70% of normal bladders showed post‐treatment recovery, indicating preservation or remodeling of normal bladder tissue following VTP.

Previous experiments on MB49 BC cells showed that VTP effectively ablated primary subcutaneous tumors.^17^ In the present study, we established a mouse orthotopic bladder model initially described by Hiles et al.^9^ VTP on this model was performed on Day 6 post‐injection when the tumor cells invaded the bladder musculature, mimicking muscle‐invasive tumor treatment. At Day 10 post‐VTP, half of the treated animals presented >95% tumor necrosis. Follow‐up of the treated animals for longer times showed tumor regrowth in 84% of the animals on Day 36 post‐treatment enforcing their termination. The last animal in the untreated control group was sacrificed on Day 14. Histologically, 36 days post‐VTP, the treatment group showed non‐damaged bladders in the presence of viable tumor cells.

Previously, it was shown that WST11‐VTP initiates an immune response and establishes prolonged immunity in animal cancer models.^6^, ^17^, ^18^ Rozenzweig et al. demonstrated that after WST11‐VTP, the populations of CD8^+^ T cells in the tumor and CD4^+^ T cells in the spleen and lungs were significantly higher in surviving animals, suggesting their pivotal role in anti‐tumor and immune‐mediated VTP effects.^19^ Previously, we found that VTP significantly increased the infiltration of CD11b^+^ myeloid cells and M2 macrophages and the expression of CRF1 in a prostate cancer model.^20^ The combination of VTP with antiCSF1R reduced MDSCs and increased the infiltration of CD8^+^ T cells, which was related to decreased tumor progression and improved survival. VTP treatment with a PD1 inhibitor/OX40 agonist on an MB49 mouse model also increases CD8^+^ T cells in the tumor.^21^ Treatment of renal tumors with VTP in combination with systemic PD1/PDL1 pathway inhibition resulted in tumor regression, prolonged survival, and decreased lung metastasis development, which was associated with an increased ratio of CD8^+^: regulatory T cells and increased T cell infiltration at sites of lung metastases.^22^

The overall tumor‐free animal survival in this study was significantly lower than that in subcutaneous animal models, most likely because of the short illumination periods of intermittent light application. However, acute tumor ablation (94%) and a similar overall immune response support the use of the subcutaneous model to determine optimal treatment parameters. In the present study, we used an orthotopic mouse bladder model which is too small for a conclusion about clinical relevance. To achieve a greater clinical relevance VTP was shown to be safe and effective when applied in the oncopig bladder model (Aulitzky A., unpublished data), developed in our laboratory.^23^ VTP on a normal pig bladder showed that tissue ablation reached adequate depth.^24^

## CONCLUSION

In summary, our study suggests that WST11‐VTP treatment of bladder tumors in a mouse model results in tumor ablation and an anti‐tumor immunity boost while preserving normal bladder tissue. In addition, VTP of muscle‐invasive bladder tumors decreased tumor growth and prolonged survival compared to the untreated control. These effects correlated with an increase in CD8^+^/CD4^+^ T cells and NK cells in the tumor. This finding suggests that combinations with immunotherapy may enhance cure.

## AUTHOR CONTRIBUTIONS

**Jie Chen, Natalia Kudinova**: data curation, methodology, investigation, and writing‐review. **Jasmine Thomas, Karan Nagar, Lucas Nogueira:** Data curation, methodology. **Rebecca Dubrovsky, Kwanghee Kim, Avigdor Scherz, Jonathan Coleman**: Review and editing. All the authors have read and approved the final manuscript.

## FUNDING INFORMATION

This work was supported by the Thompson Family Foundation to Jonathan Coleman.

## CONFLICT OF INTEREST STATEMENT

The authors declare no conflict of interest.

## Data Availability

The data generated in this study are available upon request from the corresponding author.
